# Endoscopic submucosal dissection versus surgery for T1b esophageal carcinoma: a single-center retrospective study

**DOI:** 10.1007/s00432-024-05724-3

**Published:** 2024-05-09

**Authors:** Ting Zhang, Hanying Wang, Tian Jin, Zeyu Wu, Xiuqing Li, Qide Zhang

**Affiliations:** 1 Department of Endoscopy, Affiliated Hospital of Nanjing University of Chinese Medicine, Nanjing, Jiangsu People’s Republic of China; 2 Department of Pathology, Affiliated Hospital of Nanjing University of Chinese Medicine, Nanjing, Jiangsu People’s Republic of China

**Keywords:** Superficial esophageal carcinoma, Endoscopic submucosal dissection, Surgery

## Abstract

**Introduction:**

Endoscopic submucosal dissection (ESD) is a preferred treatment option for superficial esophageal squamous cell carcinoma (SESCC). However, only few studies compared long-term survival outcomes of ESD with surgery, especially for T1b SESCC. This study compared the overall survival (OS), disease-free survival (DSS), recurrence-free survival (RFS), and complication rates of both, to evaluate the value of ESD in patients with T1b SESCC.

**Methods:**

We reviewed patients who underwent ESD (*n* = 47) or surgery (*n* = 73) for T1b SESCC at Affiliated Hospital of Nanjing University of Chinese Medicine from 2009 to 2021. To increase the precision of our results interpretation, subgroups were analyzed according to the depth of tumor invasion and elderly people.

**Results:**

In the ESD and surgery groups, the overall mortality rates were 0/100 and 12.3/100 person years, incidence rates of recurrence were 2.13/100 and 11/100 person years, respectively. Kaplan–Meier survival analysis revealed no significant different in OS, DSS and RFS. Charlson comorbidity index (CCI) and depth of submucosal invasion were identified as risk factors for cancer recurrence in multivariate analysis. For elderly people, no significant differences were found in OS, DSS and RFS between different treatments.

**Conclusion:**

ESD are related to lower complication rates and shorter hospital stay than surgery in long-term outcomes for patients with pT1b SESCC. But in pT1b-SM2 patients, we still need long-term follow-up.

## Introduction

Esophageal cancer (EC) is one of the most common cancers of the digestive tract worldwide with an estimated 455,800 cases in China, making it the fourth leading cause of death in 2012 (Domper Arnal et al. 2015). Esophagectomy is considered the standard of care for patients with superficial esophageal squamous cell carcinoma (SESCC), but this procedure is associated with high morbidity and mortality rates, especially in patients at high surgical risk (Atkins et al. 2004). With the development of endoscopic techniques, endoscopic submucosal dissection (ESD) has become an effective alternative treatment for SESCC without distant metastasis. According to the Japanese EC guidelines, SESCC for MM or SM1 lesions with no clinical evidence of lymph node metastasis (LNM) can be treated with ESD. Treatment of pT1-SM2 lesions remains controversial because a few studies have shown that 50% of such lesions are associated with metastasis (Japan Esophageal S 2017; Committee et al. 2015). Follow-up is mandatory for pT1b SM1 and SM2 lesions. In particular, the long-term outcomes of pT1b-SM2 lesions are still unknown in China. This study was performed to retrospectively compare the safety, efficacy, and long-term outcomes of ESD versus surgery in patients with pT1b SESCC.

## Methods

### Study population

**Fig. 1:**
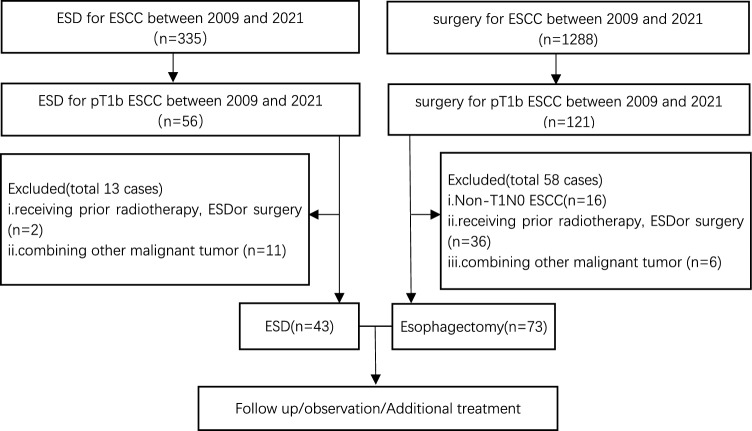
Flow diagram of patients who underwent ESD or surgery for T1b SESCC.* ESD* endoscopic submucosal dissection,* SCC* squamous cell carcinoma,* SESCC* superficial esophageal squamous cell

From Match 2009 to May 2021, we examined patients who received ESD and surgery for pT1b SESCC at Jiangsu Provincial Hospital of Chinese Medicine, which is connected to Nanjing University of Chinese Medicine. The following were the inclusion criteria: (i) patients in the ESD group and surgery group who were clinically staged T1bN0M0 (cN0M0). The following were the exclusion criteria: (i) individuals who had surgery, radiation, or ESD in the past; (ii) those who also had another malignant tumor; (iii) those who previously underwent radiation therapy or chemotherapy for the underlying illness (Fig. 1).

The yearly count of cases for every surgery listed in Table 1. The study was approved by the Ethics Committee. All patients gave their written informed consent for the retrospective chart review.

**Table 1:** Characteristics of demographic, pathological parameters, treatment and outcome of T1b SESCC

	Overall			Age over 70 years		
	ESD (*N* = 47)	Surgery (*N* = 73)	*P* value	ESD (*N* = 15)	Surgery (*N* = 19)	*P* value
Age (years), mean ± SD	66.17 ± 6.82	64.38 ± 7.64	0.19	73.33 ± 3.37	73.47 ± 3.27	0.90
Male	32	54	0.62	13	11	0.15
CCI, *n* (%)			0.41			0.34
0	41	67		11	16	
1	6	5		4	2	
≥ 2	0	1		0	1	
Family history, *n* (%)	2(4.65)	4(5.48)	1	0	0	
Multiple lesions, *n* (%)	9(19.1)	7(9.59)	0.22	4	2	0.44
Lesion location, *n* (%)			0.42			0.76
Upper	12(25.5)	15(20.5)		3	3	
Middle	20(42.5)	40(54.8)		6	10	
Lower	15(31.9)	18(24.7)		6	6	
Macroscopic type (%)			< 0.01			< 0.01
Type 0–I	1	38		1	10	
Type 0–II	46	29		14	7	
Type 0–IIa	15	5		6	2	
Type 0–IIb	15	6		4	2	
Type 0–IIc	3	5		1	1	
Type 0-IIa + IIb	1	4		1	0	
Type 0-IIb + IIc	3	4		1	1	
Type 0-IIa + IIc	9	5		1	1	
Type 0–III	0	6		0	2	
Tumor size (cm) mean ± SD	1.95 ± 1.29	2.06 ± 1.04	0.61	1.79 ± 0.97	2.01 ± 0.98	0.51
Histology, *n* (%)			0.21			0.32
Well-moderately differentiated	39(83.0)	53(72.6)		12 (80.0)	11(57.89)	
Poorly differentiated	8(17.0)	20(27.4)		3 (20.0)	8(42.11)	
Depth of invasion, *n* (%)			0.13			0.32
T1b-SM1	14(29.8)	34(46.6)		3(20.0)	8(42.11)	
T1b-SM2	33(70.2)	39(53.4)		12(80.0)	11(57.89)	
LVI, *n* (%)	4	5	1.0	0	1(5.26)	1.0
Resection margin positive, *n* (%)	2(4.2)	0	0.3	0	0	
R0 resection, *n* (%)	42(89.4)	68(93.1)	0.46	15(100)	18 (94.7)	0.56
Complications, *n* (%)	10(21.3)	34(46.6)	0.005	4	12	
Bleeding	3	5	1.0	1	1	1.0
Perforation	0	1	1.0	0	0	
Pulmonary complication	1	3	0.94	1	1	1.0
Stricture	6(12.8)	10(13.7)	0.88	2	5	0.62
Anastomotic leakage	0	7	0.07	0	2	0.57
Vascular thromboembolism	0	1	1	0	0	
Wound dehiscence	0	6		0	2	
Ileus	0	1		0	1	
Clavien–Dindo classification, *n* (%)			0.04			0.51
Non/grade I	31(66.0)	40(54.8)		9(60.0)	7(36.84)	
Grade II	4(8.51)	18(24.7)		3(20.0)	6(31.58)	
Grade IIIa	8(17.0)	11(15.1)		3(20.0)	5(26.32)	
Grade IIIb or more	0	4(5.48)		0	1(5.26)	
Post-procedure hospital stays	4.62 ± 1.28	12.7 ± 2.89	< 0.01	4.93 ± 1.33	12.95 ± 9.74	< 0.01
Additional therapy, *n* (%)			0.31			0.89
Surgery	10(21.3)	1(1.37)		13(86.67)	15(18.95)	
Radiochemotherapy	6(12.8)	16(21.9)		2(13.33)	4(21.05)	
Follow-up duration, months	47.34 ± 22.02	57.82 ± 42.90	0.12	41.13 ± 17.23	43.00 ± 32.99	0.84
All-cause mortality, *n* (%)	1	9(12.3)	0.09	0	1(5.26)	1.0
Disease-specific mortality, *n* (%)	1	5	0.40	0	1(5.26)	1.0
Recurrence/metastasis, *n* (%)	1(2.13)	8(11.0)	0.24	1(6.67)	2(10.53)	1.0

### Pretreatment evaluation

A detailed assessment, endoscopic examination, endoscopic ultrasonography (EUS), and chest/abdominal enhanced computed tomography (CT) were performed on every patient in both the groups. To determine the extent of tumor invasion and lymph node, EUS was examined. To find potential lymph nodes or distant metastases, CT was utilized.

The patients had ESD even if postoperative pathology was staged TN0M0T1b, because preoperative magnifying endoscopy, EUS, CT, and other relevant imaging modalities were limited in assessing the degree of infiltration and lymph node metastases of superficial esophageal cancer.

### Procedure and histologic evaluation

Expert endoscopists carried out esophageal ESDs utilizing a standard method and intratracheal intubation anesthesia with carbon dioxide insufflation (Fujishiro et al. 2009). In this investigation, a dual knife (KD-650Q, Olympus, Japan) and a standard endoscope (GIF-Q260J, Olympus Optical, Tokyo, Japan) with a waterjet system were employed. On a board, the ESD specimen was carefully spread out and secured with pins. Hematoxylin and eosin staining and fixation with 10% formalin were followed by histological assessment on 2-mm thick sections. Sections 4 mm thick were used to assess the surgical specimens following standard fixation. Tumor histology, grade of differentiation, size, invasion depth, lympho-vascular invasion (LVI), and presence of tumor in resection margin were assessed for pathologic specimens in both groups. The eighth edition of the American Joint Committee on Cancer staging guidelines for esophageal malignancies was used to determine staging. According to the Japan Esophageal Society guideline, the depth of submucosal invasion was classified into 2 groups: SM1 (submucosa invasion ≤ 200 μm from the muscularis mucosae) and SM2 (submucosa invasion > 200 μm from the muscularis mucosae) (Japan Esophageal S^2017^). Multiple esophageal lesions were present in seven individuals in the surgery group and nine patients in the ESD group.

### Follow-up

Post-treatment surveillance of recurrence was intensively performed. Adjuvant therapy, which includes extra surgical procedures, radiation therapy, and/or chemotherapy, was taken into consideration for patients with positive margins, as well as ESD patients who had poorly differentiated lesions, lympho-vascular invasion or SM2 lesions. Nevertheless, the patient's physical state, anticipated life expectancy, and personal preferences all played a role in the final choice. For 5 years, the surgical group underwent annual endoscopic exams and chest CT scans. Endoscopic evaluations were carried out for the ESD group after 6 months, 1 year, 18 months, 2 years, and annual year; thereafter, annual chest CT scans were conducted. The majority of the follow-up data came from medical records. The phone was used to find out the most recent status of patients who had switched hospitals.

### Investigated variables and outcomes

The following variables were investigated: age, sex, CCI, pathological information (tumor location, size, grade of differentiation, invasion depth, LVI, and tumor presence in resection margin), hospital stays, post-operation adverse event, follow-up period, pattern of cancer recurrence, and cause of death.

OS, DSS and RFS were evaluated. OS was defined as the period from treatment to death from all causes. DSS was defined as the period from treatment to death from esophageal cancer. RFS was defined as the period from treatment to recurrence of esophageal cancer. Follow-up periods were calculated from the date of ESD or surgery. Survival was assessed on the most recent outpatient visit or telephone evaluation date of January 30, 2022. Time to recurrence was calculated from the date of ESD or surgery to the time of the latest endoscopic evaluation in our hospital or another one.

Reviews of adverse events from both the early and late stage of treatment were conducted. Events that happened within 30 days of treatment were classified as early adverse events, while events that happened more than 30 days later were classified as late adverse events. ESD side effects included perforation, bleeding that required transfusion, and stricture that required treatment. Postoperative adverse effects included pneumonia and respiratory insufficiency, arrhythmia, acute kidney injury, and wound infection/dehiscence, anastomotic leakage, hemorrhage, fistulization, and stricture development in the surgery. Acute negative events were categorized using the Clavien–Dindo system. In the event that a tumor recurrence was discovered during follow-up, the location was noted and divided into two categories: distant and locoregional.

### Statistical analyses

To compare categorical variables, the Pearson *χ*^2^ test or Fisher exact test was used. Comparison of continuous variables was performed using the Student* t* test or the Mann–Whitney *U* test. The Kaplan–Meier method was used for survival analysis. Statistically significant variables were set at* P* value < 0.05 (2 sided). For these, analyses were performed in SPSS version 22.0 for Windows.

## Results

### Patients’ clinicopathologic characteristics

Clinicopathologic characteristics of all patients are shown in Table 1. 47 patients underwent ESD and 73 patients underwent surgery. The ESD group showed higher proportion of well-differentiated histology (83.0% vs 72.6%, *p* = 0.18), higher probability of tumor-positive resection margin (4.2% vs 0%, *p* = 0.3) and higher rate of rescue surgery (21.3% vs 1.37%, *p* = 0.0002). The surgery group showed higher rate of chemo/radiation therapy (21.9% vs 12.8%, *p* = 0.015). There was no difference in age, sex, CCI, tumor location, and the presence of LVI in either group.

### Survival outcome and cancer recurrence

**Fig. 2:**
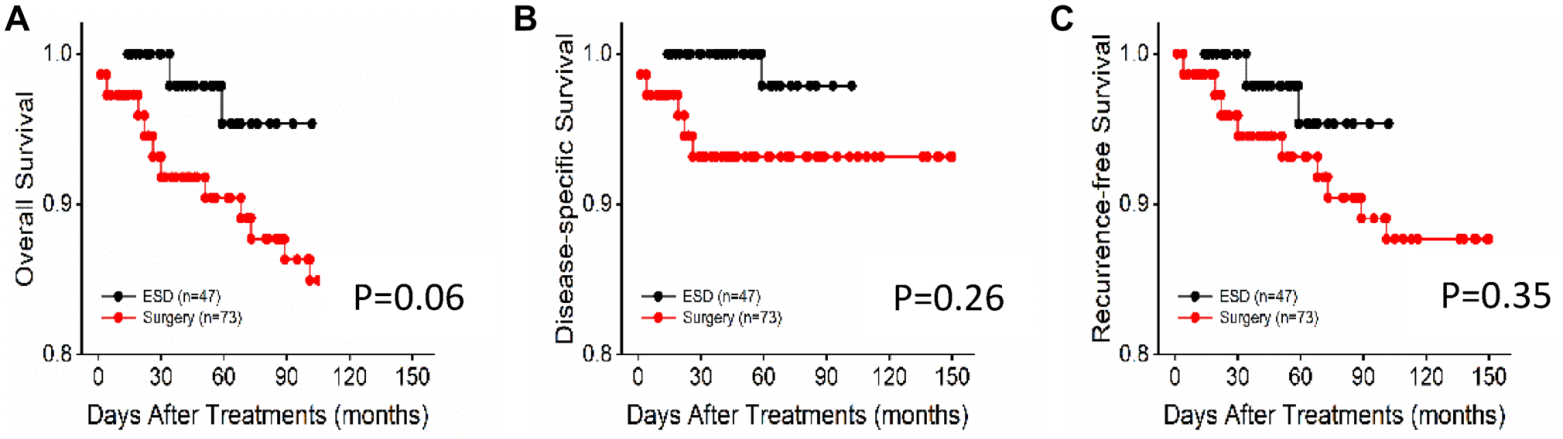
Kaplan–Meier survival curves for ESD and surgery groups in T1b ESCC. **A** Overall survival. **B** Disease-specific survival. **C** Recurrence-free survival. Red line ESD group (group 1), Black line surgery group (group 2)

Table 1 also shows the comparison of OS and recurrence in the two groups. The median follow-up periods for survival were 47 months (interquartile range 14–102 months) in the ESD group and 63 months (interquartile range 6–150 months) in the surgery group. The overall mortality rate was 0/100 and 12.3/100 person years in the both, respectively. Incidence rates of recurrence were 2.13/100 person years in the ESD group and 11/100 person years in the surgery group. Kaplan–Meier survival analysis revealed no significant difference in OS, DSS and RFS (Fig. 2).

### Hospital stay and adverse events

Table 1 also shows the length of hospital stay and adverse events of both treatments. The ESD group showed shorter hospital stay (median 4.62 days vs 12.7 days) and lower overall adverse events (21.3% vs 46.6%, *p* = 0.005) than the surgery group. Stricture requiring intervention was the most common cause of late adverse events in both groups (12.8% vs 13.7%, *p* = 0.88).

### Predictors of OS and cancer recurrence

Cox proportional hazards regression analysis was used for finding the association factor between OS and cancer recurrence. On multivariate analysis, complications were associated with poor survival outcomes (complications, hazard ratio [HR] 3.116 [1.394–6.967], *p* = 0.006. LVI and complications were identified as risk factors for cancer recurrence in multivariate analysis (LVI, HR 0.044 [0.003–0.710], *p* = 0.028; complications, HR 3.087 [1.167–8.165], *p* = 0.023). There was no statistical difference in mortality and recurrence risk according to treatment modality.

### Subgroup analysis based on the depth of tumor invasion

**Fig. 3:**
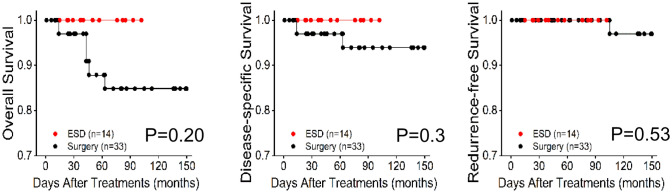
Kaplan–Meier survival curves for ESD and surgery groups in T1b-SM1 ESCC

**Fig. 4:**
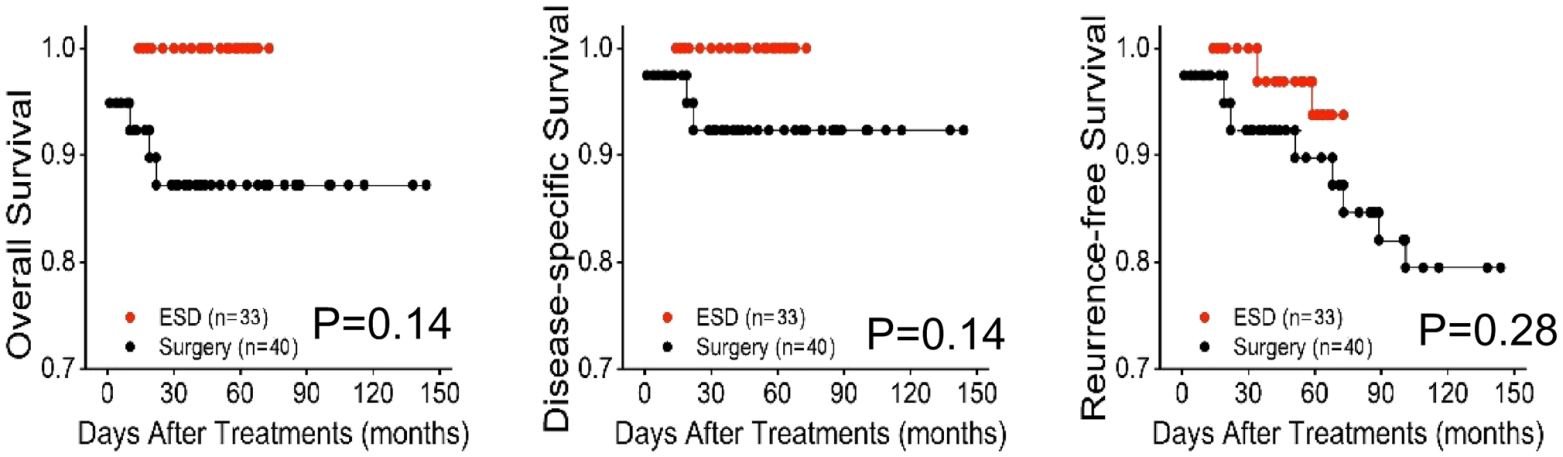
Kaplan–Meier survival curves for ESD and surgery groups in T1b-SM2 ESCC

Subgroup analysis was taken to better comprehend the benefits and risks of ESD based on the depth of tumor invasion compared to surgery. For SM2 subgroups, lesion diameter was larger in the surgery group than in the ESD group. The ESD group showed higher proportion of well-differentiated histology and CCI than another group. But all other patient characteristics and pathological parameters were comparable between the two groups (Table 2). For the type T1b-SM1 and SM2, possibility of R0 resection and use of additional therapy were similar between the two groups (Table 2). For both two subgroups, ESD patients had shorter procedure duration, shorter post-procedure hospital stay, and lower incidence of severe complications (Table 2). The overall mortality rate of ESD group was 0/100 person years and 3/100 person years in the type T1b-SM1 and SM2, respectively. Incidence rates of recurrence of ESD group were 0/100 person years in the type T1b-SM1 and 3/100 person years type T1b-SM2. The overall mortality rate of surgery group was 12.1/100 person years and 12.5/100 person years in the type T1b-SM1 and SM2, respectively. Incidence rates of recurrence of ESD group were 3/100 person years in the type T1b-SM1 and 20/100 person years type T1b-SM2.In all, for those two subgroups, no significant differences were made in oncologic outcomes between the ESD and surgery groups (Table 2). In addition, Kaplan–Meier survival analysis revealed no significant differences in OS, DSS and RFS in T1b-SM1 and T1b-SM2 subgroups between ESD and surgery treatment (Figs. 3, 4).

**Table 2:** Characteristics of demographic, pathological parameters, treatment and outcome before and after propensity score-matched cohort

	SM1			SM2		
	ESD (*N* = 14)	Surgery (*N* = 33)	*P* value	ESD (*N* = 33)	Surgery (*N* = 40)	*P* value
Age(years), mean ± SD	64.14 ± 9.47	64.21 ± 8.18	0.98	67.03 ± 5.28	64.53 ± 7.26	0.10
Male	9 (64.29)	27 (81.82)	0.36	23 (69.7)	27 (67.5)	1.0
Charlson comorbidity index, *n* (%)			0.43			0.64
0	12 (85.71)	32 (96.97)		29 (87.88)	35 (87.50)	
1	2 (14.29)	1 (3.03)		4 (12.12)	4 (10.00)	
≥ 2	0	0		1	1 (2.50)	
Family history, *n* (%)	1 (7.14)	1 (3.03)	1.0	1 (3.03)	3 (7.5)	0.75
Multiple lesions, *n* (%)	4 (28.57)	2 (6.06)	0.1	5 (15.15)	5 (12.5)	1.0
Lesion location, *n* (%)			0.56			0.39
Upper	4 (28.57)	5 (15.15)		8 (24.24)	10 (25.00)	
Middle	7 (50.0)	19 (57.58)		13 (39.39)	21 (52.50)	
Lower	3 (21.43)	9 (27.27)		12 (36.36)	9 (22.50)	
Macroscopic type (%)			< 0.01			< 0.01
Type 0–I	0	16		1 (3.03)	22 (55.00)	
Type 0–II	14	16		32 (96.97)	13 (32.50)	
Type 0–IIa	3	3		12	2	
Type 0–IIb	8	5		7	1	
Type 0–IIc	0	3		3	2	
Type 0-IIa + IIb	0	2		1	2	
Type 0-IIb + IIc	3	2		0	2	
Type 0-IIa + IIc	0	1		9	4	
Type 0–III	0	1		0	5 (12.50)	
Tumor size(cm), mean ± SD,	2.63 ± 1.87	2.04 ± 1.22	0.21	1.66 ± 0.82	2.07 ± 0.87	0.04
Histology, *n* (%)			0.43			0.05
Well-moderately differentiated	12 (85.71)	29 (87.88)		27 (81.82)	23 (57.50)	
Poorly differentiated	2 (14.29)	4 (12.12)		6 (18.18)	17 (42.50)	
LVI, *n* (%)	2 (14.29)	2 (6.06)	0.72	2 (6,06)	3 (7.50)	1.0
Resection margin positive, *n* (%)	0	0		2 (6.06)	0	0.39
R0 resection, *n* (%)	12 (85.7)	31 (96.9)	0.35	30 (90.9)	37 (92.5)	0.81
Complications, *n* (%)						
Bleeding	0	4	0.43	3	1	0.47
Perforation	0	0		0	1	1.0
Pulmonary complication	0	1	1.0	1	2	1.0
Stricture	0	6	0.22	6	4	0.5
Anastomotic leakage	0	2	0.88	0	5	0.1
Vascular thromboembolism	0	0		0	1	1.0
Wound dehiscence	0	2	0.88	0	4	0.5
Ileus	0	0		0	1	1.0
Clavien–Dindo classification, n (%)			0.2			0.04
Non/grade I	13 (92.86)	21 (63.64)		22 (66.67)	19 (47.50)	
Grade II	1 (7.14)	5 (15.15)		3 (9.09)	13 (32.50)	
Grade IIIa	0	5 (15.15)		8 (24.24)	6 (15.00)	
Grade IIIb or more	0	2 (6.06)		0	2 (5.00)	
Post-procedure hospital stays	4.36 ± 0.74	10.52 ± 4.82	< 0.01	4.73 ± 1.44	14.50 ± 10.03	< 0.01
Additional therapy, *n* (%)						
Surgery	2 (14.29)	0		8	0	
Radiochemotherapy	1 (7.14)	5 (15.15)	0.78	5 (15.15)	11 (27.50)	0.32
Follow-up duration, months	55.00 ± 28.51	62.03 ± 45.27	0.59	44.09 ± 18.17	54.35 ± 41.09	0.19
All-cause mortality, *n* (%)	0	4 (12.12)	0.43	1	5 (12.5)	0.21
Disease-specific mortality, *n* (%)	0	2 (6.06)	0.88	1	3 (7.5)	0.62
Recurrence/metastasis, *n* (%)	0	1 (3.03)	1.0	2 (6.06)	8 (20.0)	0.17

### Subgroup analysis based on elderly people

**Fig. 5:**
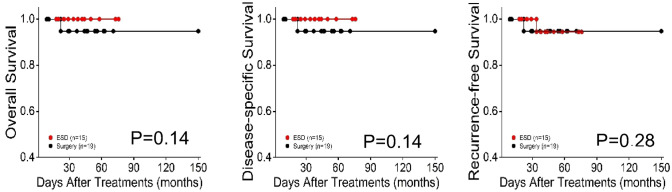
Kaplan–Meier survival curves for ESD and surgery groups in T1b ESCC on elderly people

Between the ESD and surgery groups, mean follow-up duration was comparable (P = 0.89). At the end of follow-up, no significant differences were found in all-cause mortality rate, disease-specific mortality, and recurrence and/or metastasis rate (Table 1). Kaplan–Meier analysis showed no significant differences in OS (*p* = 0.14), DSS (*p* = 0.14) and RFS (*p* = 0.28) (Fig. 5).

### Additional treatments for non-R0-resected patients

**Fig. 6:**
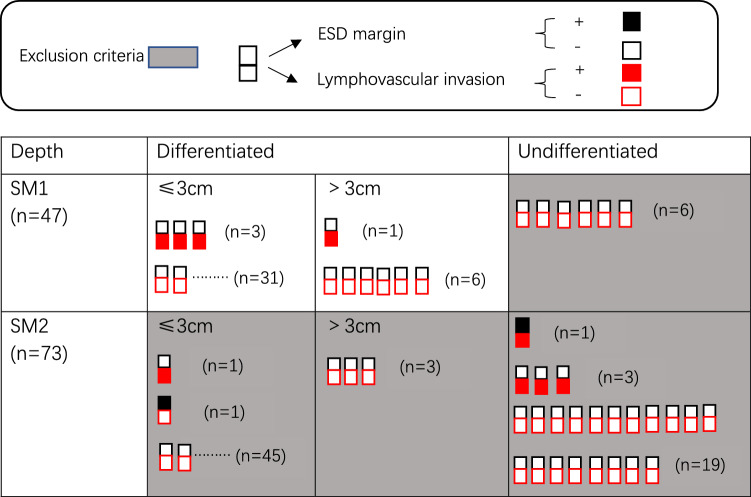
Postoperative pathology of ESD for T1b SESCC

Post-procedure pathological analysis showed that 10 patients had non-R0 resection; among the 5 non-R0 resection patients in the ESD group, 2 received rescue surgery and 1 received additional radiochemotherapy. Among the 2 patients who received rescue surgery, postoperative pathological result showed no residual tumor tissue or LNM. In the surgery group, 2 patients with non-R0 resection received radiochemotherapy (Fig. 6). Chemoradiotherapy as additional treatment were taken in 16 patients of surgery group and 6 patients of ESD group, with the overall survival rate of 81.3% (13/16) and 83.3% (5/6).

## Discussion

Surgery was historically the main treatment modality for EC, but it has been associated with high mortality and complication rates. Recent advances in endoscopy have enabled detection and curative resection of early EC. The widely accepted viewpoint is that mucosal invasive carcinoma has a low incidence of LNM, whereas submucosal invasive carcinoma has a relatively high incidence of LNM (Yachida et al. 2020). The major controversy regarding ESD versus surgery centers around pT1b SESCC, therefore, we focused on this group of patients alone.

The JCOG0508 trial was a corroboratory study of the efficacy of endoscopic resection (ER) followed by chemoradiotherapy in patients with SM1/SM2 cancer. The study showed that the 3-year OS rate for all patients was 92.6% [90% confidence interval (CI), 88.5–95.2%] and that the 3-year progression-free survival rate was 89.7% (90% CI, 84.2–93.4%). Favorable results were achieved in the prophylactic chemoradiotherapy group, with a 3-year OS rate of 90.7% (90% CI, 84.0–94.7%) (Minashi et al. 2019). Our study supports the results of previous comparative studies, showing comparable long-term outcomes between ESD and surgery in patients with pT1b SESCC. In our study, ESD showed survival and recurrence rates comparable to those of surgery. Our results indicate that ESD can be performed safely for SM1/SM2 cancer. A study from Korea showed no difference between the ESD and surgical resection groups after 43 and 63 months of observation, respectively, in terms of OS, DSS, or RFS (Min et al. 2018). In our study, the survival analysis also revealed no significant different in OS, DSS, or RFS between ESD and surgery in patients with T1b SESCC. ER is, therefore, considered safer and less invasive than surgery in patients with T1b SESCC, as well as being superior in terms of lower medical cost (Zhang et al. 2019).

A subgroup analysis of T1b SESCC is necessary. One study showed that T1b tumors without histopathological high-risk markers of LNM can be endoscopically resected with a good prognosis (Graham et al. 2018). Compared with T1b-SM1, reasonable decision-making and optimal management strategies are lacking for T1b-SM2 cancer. The JCOG0502 trial showed a good 5-year survival rate of 86.5% after surgical resection in patients with T1b-SM2 cancer. Grade 3 and 4 adverse events, including anastomotic leakage, occurred in 6.3% of patients, pneumonia in 7.7%, recurrent laryngeal nerve palsy in 2.9%, and fistula in 1.9% (Oshima et al. 2022). In our study, the Kaplan–Meier survival analysis revealed no significant differences in OS, DSS, or RFS in the T1b-SM1 and T1b-SM2 subgroups between ESD and surgery. However, the SM2 group had significantly higher recurrence and metastasis rates than the SM1 group (6% vs. 0% and 20% vs. 3%, respectively). Therefore, the subgroup analysis indicates that we should determine the treatment strategy of T1b-SM2 with care.

In aging societies, choosing the optimal treatment for elderly patients with SESCC is crucial. It is difficult to define “elderly.” The Joint Committee of the Japan Gerontological Society and the Japan Geriatrics Society defined “elderly” as an age of ≥ 75 years (Ouchi et al. 2017), but in patients with EC, this age decreased to 70 to 74 years in 2014 (Global Burden of Disease Cancer Collaboratio et al. 1990). Therefore, in our study, elderly people were defined as those aged ≥ 70 years. Regarding long-term outcomes, our data demonstrate that compared with surgery, the prognosis after ESD is acceptable in elderly patients. Nevertheless, additional surgery should be considered if an elderly patient is in good health.

Surgery and chemoradiotherapy are strongly recommended as additional treatments for T1b-SM2 SESCC without vascular invasion following ER (Ishihara et al. 2020). A retrospective study of definitive chemoradiotherapy in 36 patients with cT1bN0M0 esophageal squamous cell carcinoma showed that local and metastatic recurrences were common, with a 5-year OS rate of 86% and a 5-year DFS rate of 59% (Kato et al. 2009; Murakami et al. 2015). In our study, chemoradiotherapy as additional treatment was administered to 16 patients in the surgery group and 6 patients in the ESD group, with an OS rate of 81.3% (13/16) and 83.3% (5/6), respectively. However, no large, prospective, adequately credible studies have yet been performed to compare surgical resection and chemoradiotherapy as additional treatments.

The current study has several limitations, including the relatively small number of patients, the fact that it was performed in a single institution, and its retrospective design, each of which can limit accuracy of the results. Furthermore, the preoperative management, diagnostic methods, and postoperative care were heterogeneous. Moreover, the techniques changed and the technology was improved over the study period, potentially influencing the results.

In conclusion, ESD is related to lower complication rates and shorter hospital stays than surgery with respect to the long-term outcomes for patients with pT1b SESCC. In patients with pT1b-SM2 cancer, however, long-term follow-up is still needed.

## Data Availability

The data that support the findings of this study are available from the corresponding author, upon reasonable request.
